# Morphological profiling for drug discovery in the era of deep learning

**DOI:** 10.1093/bib/bbae284

**Published:** 2024-06-17

**Authors:** Qiaosi Tang, Ranjala Ratnayake, Gustavo Seabra, Zhe Jiang, Ruogu Fang, Lina Cui, Yousong Ding, Tamer Kahveci, Jiang Bian, Chenglong Li, Hendrik Luesch, Yanjun Li

**Affiliations:** Calico Life Sciences, South San Francisco, CA 94080, United States; Department of Medicinal Chemistry, Center for Natural Products, Drug Discovery and Development, University of Florida, Gainesville, FL 32610, United States; Department of Medicinal Chemistry, Center for Natural Products, Drug Discovery and Development, University of Florida, Gainesville, FL 32610, United States; Department of Computer & Information Science & Engineering, University of Florida, Gainesville, FL 32611, United States; Department of Computer & Information Science & Engineering, University of Florida, Gainesville, FL 32611, United States; J. Crayton Pruitt Family Department of Biomedical Engineering, Herbert Wertheim College of Engineering, University of Florida, Gainesville, FL 32611, United States; Department of Medicinal Chemistry, Center for Natural Products, Drug Discovery and Development, University of Florida, Gainesville, FL 32610, United States; Department of Medicinal Chemistry, Center for Natural Products, Drug Discovery and Development, University of Florida, Gainesville, FL 32610, United States; Department of Computer & Information Science & Engineering, University of Florida, Gainesville, FL 32611, United States; Department of Health Outcomes and Biomedical Informatics, College of Medicine, University of Florida, Gainesville, FL 32611, United States; Department of Medicinal Chemistry, Center for Natural Products, Drug Discovery and Development, University of Florida, Gainesville, FL 32610, United States; Department of Medicinal Chemistry, Center for Natural Products, Drug Discovery and Development, University of Florida, Gainesville, FL 32610, United States; Department of Medicinal Chemistry, Center for Natural Products, Drug Discovery and Development, University of Florida, Gainesville, FL 32610, United States; Department of Computer & Information Science & Engineering, University of Florida, Gainesville, FL 32611, United States

**Keywords:** artificial intelligence, deep learning, morphological profiling, drug discovery

## Abstract

Morphological profiling is a valuable tool in phenotypic drug discovery. The advent of high-throughput automated imaging has enabled the capturing of a wide range of morphological features of cells or organisms in response to perturbations at the single-cell resolution. Concurrently, significant advances in machine learning and deep learning, especially in computer vision, have led to substantial improvements in analyzing large-scale high-content images at high throughput. These efforts have facilitated understanding of compound mechanism of action, drug repurposing, characterization of cell morphodynamics under perturbation, and ultimately contributing to the development of novel therapeutics. In this review, we provide a comprehensive overview of the recent advances in the field of morphological profiling. We summarize the image profiling analysis workflow, survey a broad spectrum of analysis strategies encompassing feature engineering– and deep learning–based approaches, and introduce publicly available benchmark datasets. We place a particular emphasis on the application of deep learning in this pipeline, covering cell segmentation, image representation learning, and multimodal learning. Additionally, we illuminate the application of morphological profiling in phenotypic drug discovery and highlight potential challenges and opportunities in this field.

## Introduction

**Figure 1:**
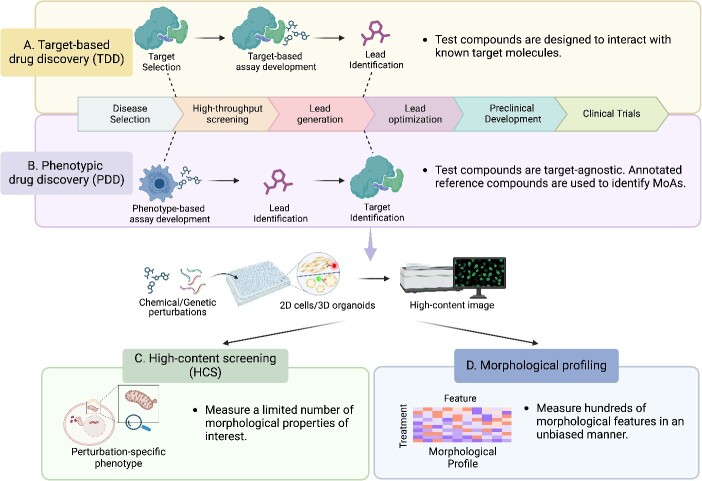
Early-stage drug discovery approaches. (A) and (B) illustrate two primary approaches in drug discovery: target-based drug discovery (TDD) and phenotypic drug discovery (PDD). (A) TDD starts with a known drug target, and a target-based assay is established to evaluate the effect of compound–target interaction. (B) In contrast, PDD employs a target-agnostic strategy, screening compounds to determine whether a phenotype of interest is induced. Due to its unbiased nature, a target identification step is required. Within the context of PDD, (C) high-content screening (HCS) and (D) morphological profiling are two commonly used approaches. The major difference is (C) HCS uses a limited number of perturbation-specific phenotypes as assay readout, whereas (D) morphological profiling obtains cellular feature representation with an unbiased approach.

Phenotypic drug discovery (PDD) plays a crucial role in drug discovery. In contrast to target-based drug discovery (TDD), where compounds are designed to interact with known target molecules, PDD takes a target-agnostic approach and focuses on phenotypic effects of compound treatment in disease-relevant biological systems [1, 2] (Fig. 1A and B). This strategy uses reference compounds with treatment class annotations to uncover previously unknown mechanisms of action (MOAs) of the test compounds. To date, PDD has made a significant contribution to the development of first-in-class drugs and the discovery of novel therapeutic opportunities [1, 2]. For example, PDD is the primary approach in natural products discovery and the basis for identification of new targets and/or MOAs. Natural products are all bioactive, and the most effective way to multiplex and assign function is through phenotypic screening, particularly by analyzing related biased and unbiased nuances from high-content imaging [3–6].

Automated microscopy and image analysis have enabled high-throughput image-based assays for PDD [1, 7]. The two approaches, namely, high-content screening (HCS) and morphological profiling, are both based on imaging experiments at a large scale, yet distinct in strategy (Fig. 1C and D). In HCS, feature measurements are limited to specific phenotypes related to perturbations. In contrast, morphological profiling (also known as image-based profiling or cytological profiling) is an unbiased approach to capture high-dimensional image data consisting of hundreds to thousands of cellular features. Conventionally, bioimage informatics tools can measure these features that span a range of morphological properties to generate phenotypic signatures for clustering and predicting perturbation bioactivity similarity [7, 8]. To this end, this approach not only provides a comprehensive morphological profile in an unbiased manner but also allows for detecting subtle or novel phenotypes.

As a dominant technique in artificial intelligence (AI), deep learning uses deep neural networks to learn representations from raw data format in a data-driven manner, often without the needs of feature engineering [9]. In the context of drug discovery, deep learning enables efficient development of novel therapeutics through various applications, such as target identification [10, 11], protein structure prediction [12, 13], drug–target interaction prediction [14–16], *de novo* drug design [17, 18], molecular property prediction [19, 20], and biological image analysis for PDD [21, 22]. In recent years, computer vision has led to a profound transformation of image-based profiling analysis in efficiency and performance, thereby expediting drug discovery and reducing computational cost [22, 23].

**Figure 2:**
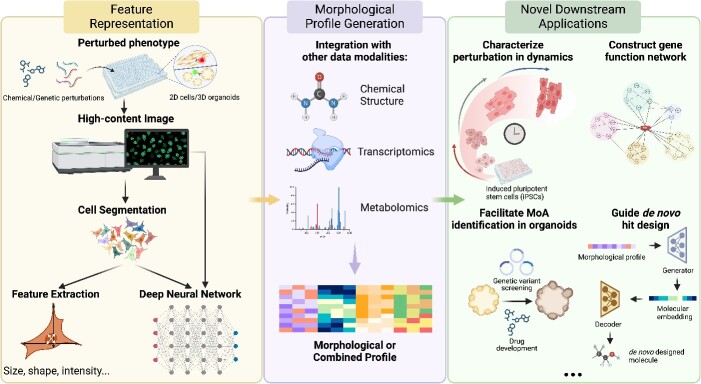
Schematic workflow of morphological profiling. After cells are perturbed and stained, fluorescent images are taken to capture cellular morphology. Single cells are detected and segmented. At the single-cell level, morphological features can be achieved with image analysis software to extract pre-defined features. Alternatively, feature vectors can be obtained through representation learning with a deep neural network. Features from single cells are subsequently aggregated into a treatment-level morphological profile. Certain deep learning models allow end-to-end learning, eliminating the need for cell segmentation. The resulting morphological profile is then applied to downstream tasks such as classification for MOA prediction and clustering for treatment association inference (left panel). Additionally, other profile modalities, such as chemical structure and transcriptomic and metabolomic profiles, can be integrated with the morphological profile to enhance downstream analysis (middle panel). Altogether, these efforts enable many novel downstream applications, such as characterizing perturbation impacts in dynamics, constructing gene function network to map genotype–phenotype relationship, identifying compound MOAs in 3D organoid model, and guiding *de novo* hit design (right panel).

In this review, we aim to provide a comprehensive overview of the extant computational approaches employed in morphological profiling with a particular emphasis on the deep learning applications. We primarily focus on the analytical pipeline of Cell Painting high-content image data given its wide application in academic research and pharmaceutical industry. We start with an introduction of Cell Painting image analysis workflow with conventional feature-engineering approach (also known as ‘handcrafted’ representation). For the major focus, we provide a thorough summary of recently proposed deep learning approaches in advancing this analytical pipeline, including microscopic image cell segmentation, representation learning from high-content fluorescent images, and multimodal learning to integrate chemical structure and omics data for MOA prediction. With concrete examples in cutting-edge applications, we conclude with our perspectives on future directions in advancing morphological profiling with deep learning solutions (Fig. 2).

## Deep learning in morphological profiling analytical pipeline

### Cell Painting and benchmark datasets

A state-of-the-art assay for morphological profiling is known as Cell Painting [24]. The canonical protocol on adherent monolayer cells uses six fluorescent dyes to characterize eight cellular components or organelles and images the fixed and stained cells in five channels on a high-throughput microscope [25]. Recent optimization efforts have further improved the assay’s capability in phenotype detection [26]. Whereas canonical Cell Painting captures cellular morphology in snapshots, technical advances now enable live-cell imaging, such as using reporter cell lines that carry organelle or pathway marker with fluorescent tag. This allows for capturing morphological profiles in dynamics [27].

Over the past decade, morphological profiling efforts from academia and pharmaceutical industry have produced several publicly available Cell Painting datasets. These include (i) the Broad Bioimage Benchmark Collection (BBBC) with compound and genetic perturbations [28–30], (ii) The Image Data Resource (IDR) with both HCS images and time-lapse images [31], (iii) the RxRx datasets released from Recursion with compounds, genetic and viral transduction perturbations, and (iv) the CytoImageNet dataset curated from 40 openly available and weakly labeled microscopy images [32]. Notably, the Joint Undertaking in Morphological Profiling Cell Painting (JUMP-CP) Consortium has recently been established as the largest public reference Cell Painting dataset [33], including images from more than 116 000 chemical perturbations and over 15 000 genetic perturbations on human osteosarcoma cells (U2OS), which were systematically acquired from 12 data-generating centers [33]. A subset of the JUMP-CP Consortium, cpg0016-jump, has been used in a recent benchmark study to evaluate self-supervised learning (SSL) methods and feature-based approaches [23]. This dataset includes single-source (data generated from a single laboratory) training set of 391 815 Cell Painting images from 35 892 compound treatments, and multisource (data generated from multiple laboratories) training set of 564 272 images from 10 057 compounds. The evaluation set includes 33 962 single-source images and 75 545 multisource images [23]. The curation of this dataset not only allows for assessing model performance using biological labels but also enables evaluation of batch effect handling [23]. An extension to this dataset, labeled CPJUMP1, has been curated to include pairs of chemical and genetic perturbations that both target the same genes in the settings of U2OS and human lung carcinoma epithelial cells (A549) [34]. This dataset consists of approximately 3 million Cell Painting images along with the feature-based profiles from 75 million single cells are well-level aggregated profiles. This unique dataset of paired annotated chemical and genetic perturbations allow for investigating gene–compound relationship [34]. These public reference datasets have been broadly used to train machine learning and deep learning models for compound bioactivity prediction and image representation learning for feature embedding. Details of these datasets are summarized in Table 1.

**Table 1:** Publicly available cellular microscopic image datasets for model training and evaluation

Data set	Description	URL	Reference
The Broad Bioimage Benchmark Collection (BBBC)	A collection of image datasets from image-based profiling and other assays annotated with different types of ground truth.	https://bbbc.broadinstitute.org/image_sets	Ljosa 2013 [28]
Recursion datasets (RxRx)	Image datasets with different perturbation modalities such as genetic, small-molecule and viral infection perturbations.	https://www.rxrx.ai/	Sypetkowski 2023 [146]
Image Data Resource (IDR)	A public repository of datasets from image-based assays.	https://idr.openmicroscopy.org/cell/	Williams 2017 [31]
JUMP Cell Painting datasets (JUMP-CP)	A multi-center image dataset of U2OS cells under genetic and compound perturbations.	https://registry.opendata.aws/cellpainting-gallery/	Chandrasekaran 2023 [33]
CPJUMP1	An image dataset of matched chemical and genetic perturbations targeting the same genes in U2OS and A549 cells.	https://broad.io/neurips-cpjump1	Chandrasekaran 2022 [34]
CytoImageNet	A dataset curated from the above publicly available microscopic images with weak labels for bioimage transfer learning.	https://www.kaggle.com/datasets/stanleyhua/cytoimagenet	Hua 2021 [32]

Among the above-mentioned datasets, the BBBC021 dataset [35] is the most commonly used benchmark to evaluate the performance of deep learning methods. This dataset, publicly available from the Broad Bioimage Benchmark Collection [28], includes Cell Painting images of human MCF-7 breast cancer cells treated with 113 compounds at eight concentrations. Most of the representation learning methods (section Representation Learning for Morphological Profiling) were compared on a subset of 103 treatments from 38 compounds. These compounds have been manually annotated with one of 12 MOAs as the ground truth. The effectiveness of different MOA prediction methods is assessed using the following evaluation metrics:

- NSC (Not-Same-Compound matching accuracy): In the NSC setting, all profiles of a test compound are deliberately excluded during the training phase and the model is tasked to predict the excluded profiles’ treatment. After generating the representation of the excluded profile, the treatment prediction is typically conducted using a 1-Nearest-Neighbor (1-NN) classifier, which assigns the test compound to its nearest neighbor within the feature space of the training compounds. This metric is to evaluate the model’s capacity to adequately generalize and correctly infer a new compound’s treatment class when its MOA is unknown [36].
- NSCB (Not-Same-Compound-and-Batch matching accuracy): NSCB serves as a more stringent metric compared to NSC. In addition to the constraints in NSC, profiles of the same experimental batch are also excluded during training. This metric enables a more robust evaluation of model’s performance and generalizability across different experiment conditions and batch settings. It can reflect the impact of batch effects and other confounding factors [37].
- Drop: Drop is calculated by subtracting NSCB from NSC. Ideally, performance drop should not be observed. The larger this metric value is, the more substantial the batch effect is [38].

### An overview of image-based profiling data analysis

**Figure 3:**
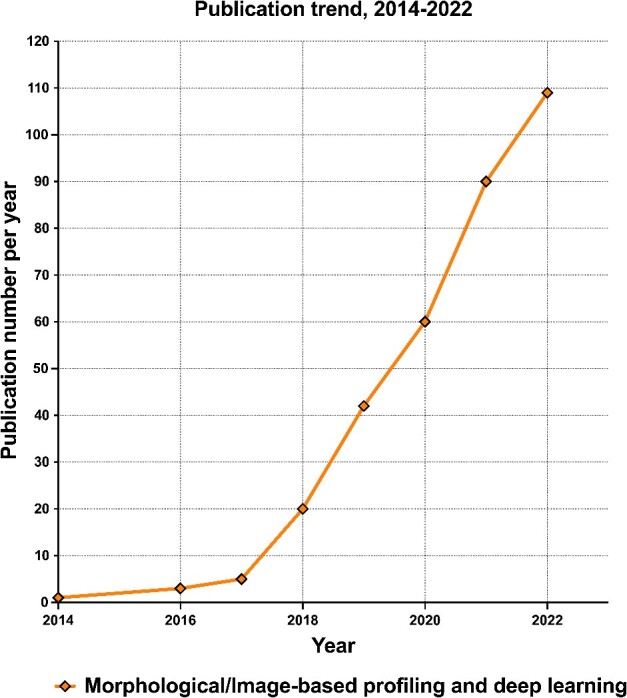
Recent publication trend of morphological profiling with deep learning. Pubmed trend demonstrates a growing number of indexed publications on morphological profiling with deep learning, including the keywords ‘deep learning’ with ‘morphological profiling’ or ‘image-based profiling.’ This trend is analyzed from 2014 to 2022.

**Figure 4:**
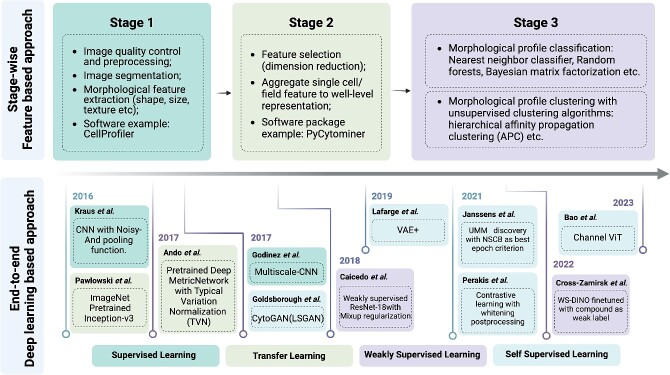
An overview of key methods and the state-of-the-art approaches in morphological profiling data analysis. Cellular images from morphological profiling assays can be analyzed using two approaches: stage-wise feature-based (top panel) and end-to-end deep learning-based (bottom panel). In the feature-based approach, image data are analyzed in four sequential stages: Stage 1 involves image preprocessing, single-cell segmentation, and feature extraction, Stage 2 aggregates cell-level features into well-level or treatment level profiles, and Stage 3 classifies each profile into the corresponding treatment class and clusters each profile based on phenotypic similarity. In contrast, deep learning–based approaches perform analysis in an end-to-end fashion with different learning paradigms. We illustrate these state-of-the-art approaches on a timeline to highlight their development.

An accurate, efficient, and generalizable imaging data analysis workflow is critical for morphological profiling. Established methods and best practices have been comprehensively documented [39–43]. However, the past few years have witnessed significant strides in the application of deep learning approaches (Fig. 3). In this section, we present an overview of the critical stages in morphological profiling data analysis, with particular emphasis on deep learning advances (Fig. 4).

#### Stage 1: Feature representation

Measuring variations in cell morphology upon perturbation relies on generating effective representations for cellular images. Conventionally, this task is implemented by feature engineering approaches. Bioimaging software like CellProfiler is commonly used to extract predefined features such as cell shape, size, and texture from fluorescent microscopy images [44]. In addition to CellProfiler, we have also summarized other open-source image analysis software and tools in Table 2. While this approach provides biologically insightful results, it requires image preprocessing and manual adjustment of parameters for every new experiment setup [39, 41]. Also, single-cell segmentation is typically required, which will be described in detail in section Deep Learning–Facilitated Cell Segmentation for Image Analysis.

**Table 2:** A selection of open-sourced image analysis tools

Tools	Website	Function
AGAVE	https://www.allencell.org/pathtrace-rendering.html	3D volume image viewer.
AICSImageIO	https://github.com/AllenCellModeling/aicsimageio	Python module for image reading, writing, and metadata conversion.
Aydin	https://github.com/royerlab/aydin	Python module for image denoising.
Bio-Formats	https://www.openmicroscopy.org/bio-formats/	Software for reading and writing image data and metadata.
BioImageIO	https://bioimage.io/#/	Deep learning model repository for image segmentation
Cellpose	https://www.cellpose.org/	Deep learning model for image segmentation.
CellProfiler	https://cellprofiler.org/	Software for automated feature extraction on large-scale image dataset.
CLIJ	https://clij.github.io/	GPU-accelerated image processing library for Fiji/ImageJ and Icy.
CytoMAP	https://gitlab.com/gernerlab/cytomap	Software for spatial analysis of segmented cell.
Cytomine	https://cytomine.com/	Web platform that allows for collaborative analysis of large biomedical image collections.
Fiji/ImageJ	https://fiji.sc/	Software for biological image analysis with many plugins.
Icy	https://icy.bioimageanalysis.org/	Software for biological image analysis.
ilastik	https://www.ilastik.org/	Interactive tool for image segmentation, classification, and analysis.
MIB	http://mib.helsinki.fi/	Software for multi-dimensional image processing, segmentation, and visualization.
Napari	https://napari.org/stable/index.html	Interactive image viewer for multi-dimensional image in Python.
Orbit	https://www.orbit.bio/	Whole slide image analysis software for digital pathology.
QuPath	https://qupath.github.io/	Whole-slide image analysis software for digital pathology.
Scikit-image	https://scikit-image.org/	Python module for image processing.
StarDist	https://github.com/stardist/stardist	Deep learning model for image segmentation as a Python module and ImageJ/Fiji plugin.

Alternatively, deep neural networks such as pre-trained convolutional neural networks (CNNs) can learn representation directly from a full-field microscopy image without the need for single-cell segmentation [37, 45]. Further, generative adversarial network (GAN)–based models and variational autoencoder (VAE) framework have been proposed to improve the interpretation of cellular structural variations that drive morphological differences [46–48] and to predict morphological responses to perturbations [49]. These advances from deep learning-based analysis approaches will be further discussed in section Representation Learning for Morphological Profiling.

#### Stage 2: Morphological profile generation

Once features are extracted from single cells or field images, these measurements will be aggregated into a single feature vector for well-level (also known as treatment-level or population-level) representation. The morphological profile generated from this stage will enable downstream well-level analysis [39].

#### Stage 3: MOA annotation

With the aggregated treatment-level morphological profiles, a common machine learning task is to predict MOA or toxicity of query perturbagens based on the known morphological profiles of the reference library [40]. This is most commonly achieved by building a feature-based machine learning model such as nearest neighbor classifier, random forests, or Bayesian matrix factorization [39, 40, 50] on top of the extracted morphological profiles. With these supervised machine learning algorithms, query perturbagens can be classified into predefined, annotated classes [40]. The aggregated morphological profiles can also be used to infer treatment-level associations. This task is typically accomplished by employing hierarchical unsupervised clustering algorithms, predicted on the similarity of morphological profiles [40]. A phenotypic similarity matrix of all pair-wise similarities between morphological profiles is computed for similarity-based clustering [40].

Notably, deep learning techniques facilitate an end-to-end learning schema, integrating all the aforementioned stages into a singular, unified process. Within this framework, the phenotypic classification and clustering tasks can be directly accomplished using raw high-content images, circumventing the explicitly image feature representation, morphological profile generation, and other intermediate steps. This end-to-end learning schema will be elaborated in section Representation Learning for Morphological Profiling.

### Deep learning–facilitated cell segmentation for image analysis

Cellular object detection and segmentation is a critical yet challenging step of microscopic image analysis. Whereas classical segmentation algorithms such as thresholding and watershed have been commonly used in bioimage analysis software [51], recent advances of deep learning in computer vision have generated various image segmentation models with substantially improved performance [52]. In the 2018 Data Science Bowl, a global competition focusing on 2D nucleus segmentation from high-content images, deep learning approaches such as U-Net, Feature Pyramid Network (FPN), and Mask-Regional Convolutional Neural Network (Mask-RCNN) dominated the leaderboard, achieving state-of-the-art performance [51]. We refer the interested readers to the report of the 2018 Data Science Bowl results for details in method and performance [51]. Each of these approaches demonstrates strengths and drawbacks. Initially designed for segmenting electron microscopy images, the U-Net model uses skip connections to append feature maps of the whole input image from the encoder to the decoder. This preserves global location information and allows for accurate reconstruction by the decoder. It can provide accurate segmentation maps with limited training data [53]. Like U-Net, FPN also leverages lateral connections. However, instead of copying and concatenating feature maps from encoder to decoder, 1 × 1 convolution is applied to allow for flexible processing [54]. In contrast to fully convolutional networks (FCNs) that use the full context of the input image, Mask R-CNN works on selected Regions of Interest (ROIs) of an input image to obtain predicted class, bounding box, and segmentation mask simultaneously. This method performs well on instance segmentation tasks to handle multiple objects with complex shapes, albeit more training examples are needed compared to U-Net [55].

A common limitation of those approaches is that their performances suffer when nuclei are packed densely. To address this challenge, STARDIST was developed to predict a flexible shape representation—a star-convex polygon instead of an axis-aligned bounding box is predicted for each pixel. When benchmarked on the 2018 data science bowl dataset, STARDIST outperformed U-Net or Mask R-CNN based models for intersection over union (IoU) threshold τ < 0.75 [56]. This method has also been successfully extended for 3D nuclei segmentation (STARDIST-3D) [57]. Fully convolutional regression networks (FCRNs) represent another solution to this challenge, regressing a cell spatial density map of the image. FCRNs demonstrated superior performance at microscopic cell counting when traditional single-cell segmentation fails due to cell clumping or overlap [58]. Another object shape representation approach is proposed by the Cellpose segmentation model. This approach generates topological maps through simulated diffusion and uses human-annotated masks as ground truth. The horizontal and vertical gradients of the topological maps are then predicted to form vector fields. Through gradient tracking, pixels that converge to the same center point are assigned to the same mask [59]. With this representation approach, the Cellpose model outperformed STARDIST, Mask R-CNN, and U-Net models at all IoU thresholds on the Cell Image Library dataset [59].

Another limitation of the above-mentioned segmentation approaches is that their training process is fully supervised, thus requiring considerable amount of expert annotations. To alleviate this requirement, Hollandi *et al*. proposed nucleAlzer, which uses image style transfer approaches to generate a set of representative image–label pairs. Applying this data augmentation paradigm to the Mask R-CNN-based model improved segmentation performance on several image datasets [60].

In addition to CNN-based models, recently, a novel deep learning architecture, CellViT, was proposed for nuclei segmentation in digitized tissue samples based on Vision Transformer (ViT) [61]. In contrast to CNN-based models, ViTs allow input images with arbitrary sizes and can capture long-range dependencies given the self-attention mechanism [62]. CellViT uses a U-Net-shaped encoder–decoder network, which leverages pre-trained ViTs such as ViT_256_ [63] and Segment Anything Model [64] (SAM) as the encoder network and bridges the encoder and decoder components at multiple network depths via skip connections [61]. Although it demonstrated SOTA performance on a histological image dataset [61], it remains to be investigated whether this model can be generalized to the single-cell segmentation task for Cell Painting datasets.

### Representation learning for morphological profiling

**Figure 5:**
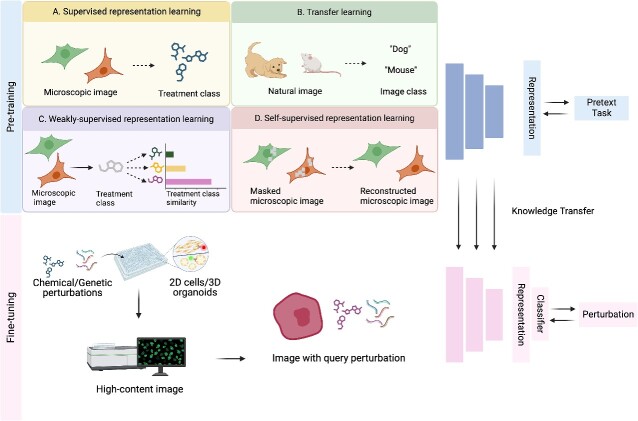
Representation learning strategies for cell morphology. At the pre-training stage, several learning strategies can be applied. (A) Supervised representation learning employs a deep neural network trained on microscopic image data with the label (treatment class). (B) Transfer learning utilizes a deep neural network initially trained on other types of annotated image data, such as natural images, to learn representations applicable to microscopic images. The pretext task is to predict the image class. (C) Weakly supervised representation learning considers the treatment labels as the weak/noisy labels. A deep neural network is trained on a pretext task to predict the treatment class of the microscopic images. The learned feature embeddings will be used to infer treatment class similarity. (D) Self-supervised representation learning utilizes the data intrinsic information for model pretraining, such as microscopic image reconstruction. These pretext tasks enhance the model's ability to learn effective representations for major tasks. Following the pretraining stage, the fine-tuning stage transfers the learned knowledge to specific downstream tasks, such as classifying query perturbations to reference perturbations for MOA inference.

Feature representation is a critical step in morphological profiling. Morphological features can either be extracted through feature-engineering approach or learned with deep neural network [65]. The former approach, however, requires manual efforts in fine-tuning software parameters per experiment setup and relies on expert knowledge to decide what phenotypic features should be measured. In contrast, deep neural networks take an unbiased approach to learn features directly from raw pixels of images and encode meaningful representations [66]. Not only do these end-to-end trained deep neural networks obviate the need for any segmentation steps but also the learned representation enables superior performance. Moreover, these networks exhibit improved transferability across different perturbation types (chemical versus genetic) and demonstrate faster pipeline processing speeds in classification tasks compared to models trained on engineered features [23, 67, 68] (Fig. 5).

#### Supervised representation learning

When extensive annotated training data is available, supervised representation learning become particularly effective [69, 70]. For example, Kraus *et al.* trained CNNs combined with multiple instances learning on annotated image dataset BBBC021 and yielded higher accuracy in treatment classification compared to the conventional feature-engineering approach [28, 36, 69, 71]. Similarly, Godinez *et al.* built a multi-scale convolutional neural network (M-CNN) based classifier, which was trained on the same annotated images [70]. This model outperformed other CNN models on classification tasks when benchmarked on several BBBC datasets.

#### Transfer learning

However, the availability of relevant annotated image data may not always be assured, and the collection of sufficient training data can be expensive and time-consuming. To that end, transfer learning of pre-trained deep neural networks becomes an alternative solution [72]. Pawlowski *et al.* for the first time proposed using ImageNet pretrained CNNs for morphological profiling feature representation, and this method achieved superior accuracy and processing speed compared to the feature engineering–based approach [73]. Similarly, Ando *et al.* proposed Deep Metric Network, a model pre-trained on ~100 million RGB consumer images, to generate embeddings for the BBBC021 image set [37]. Many other CNNs pre-trained on ImageNet have also been used to generate cell morphology embeddings [74, 75].

#### Weakly supervised representation learning

In addition to transfer learning, weakly supervised learning (WSL) approach has been proposed to train deep neural networks for learning representations of Cell Painting images [38, 76, 77]. In this learning schema, treatment or compound labels are treated as “weak” or “noisy” labels for several reasons: (i) cells may exhibit heterogeneous responses even to the same treatments; (ii) some treatments are biologically inert; however, in the context of the supervised learning setting with treatments as labels, a deep neural network is nonetheless compelled to identify differences; and (iii) different cell morphology may result from technical artifacts. Therefore, it remains uncertain whether treatment labels accurately reflect cell morphology. To leverage the weak labels, an auxiliary (or pretext) training task is introduced to train a network to classify single cell images to their corresponding treatment labels (the weak labels). Feature embeddings learned from the auxiliary classification task will subsequently be used for the major task, which is to infer the high-level associations between treatments based on similarity. In the setting of drug discovery, this allows for MOA prediction through assigning query compounds to a library of annotated reference compounds [38, 76–78]. Given that these deep neural networks are exposed to the distributions of both true biological phenotypes and confounding factors in the pretext training task, disentangling phenotypes from confounding factors is crucial to the success of this training schema. To achieve this, besides batch correction efforts (summarized in section Challenges and Outlook), a few other strategies have proven to be helpful, such as RNN-based regularization [38], convex combinations of images to generate new samples [38], and combining image datasets with strong perturbations for training [76]. Beyond representation learning with broadly used CNNs, WS-DINO from Cross-Zamirski *et al.* was proposed to learn representations using a knowledge distillation approach with ViT backbone. In this approach, global and local crops from different images under the same treatment are generated [77]. The teacher network is exposed solely to global crops, whereas the student network sees both, and the objective is to minimize the cross-entropy loss between student and teacher prediction output. Notably, in contrast to many other WSL approaches, WS-DINO does not require single cell cropping for pre-processing [77].

#### Unsupervised representation learning

Finally, unsupervised learning approaches provide another avenue for feature representation learning by identifying underlying patterns in raw data or clustering similar data into groups. Examples of such exploitable unlabeled information include whether images belong to the same treatment [79], metadata information [80], and pseudo-labels assigned by *K*-means clustering on embeddings [81]. Another strategy is to use generative models [82] such as GANs [46] or VAE framework [47, 48] to learn feature representations. They function by learning and generating new data distributions that are similar to the training data, thereby learning inherent structures and patterns within the dataset. In addition, the self-supervised learning (SSL) approach can use a pretext training task, mining the intrinsic information present in the data itself, to train a CNN capable of learning effective feature representation and use it for downstream analysis [83]. For the pretext task, Lu *et al.* proposed “paired cell inpainting,” whereby the model needs to identify protein localization from the “source” cell and predict the similar localization in the “target” cell [83]. The contrastive loss–based approach can also learn robust cell representations by training the model to bring positive example representations closer in the feature space and push the negative example representations further away from the positive ones [84]. Perakis *et al.* demonstrated that representations learned with the contrastive learning framework can be used in MOA classification task with the impressive performance on par with the transfer learning approach [37, 84]. Beyond the CNN-based model, the SSL method has also been employed to pre-train the ViT architecture, resulting in significant enhancements even in segmentation-free morphological profiling [23, 85]. In evaluations using subsets of the JUMP-CP Consortium data, the ViT architecture, trained by the recently introduced DINO SSL approach, outperformed both CellProfiler and transfer learning–based methods in several dimensions. Specifically, when trained on multisource data, this approach demonstrated the best performance in classification tasks. The resultant image representations showcased exceptional adaptability, transitioning efficaciously from chemical to genetic perturbations. Moreover, the pipeline functioned at speed 50 times faster than CellProfiler-based feature engineering workflow [23]. It is noteworthy that, unlike CNNs where local features are consolidated into aggregated vectors, ViTs preserve a more refined resolution of inputs across all network layers, and this preservation facilitates the encoding of features that are biologically meaningful at the subcellular level [85]. Notably, ChannelViT has been proposed to make a simple modification to the ViT architecture by constructing patch tokens independently from each input channel and includes a learnable channel embedding. These modifications improve model reasoning across channels, such that the model can generalize efficiently even when limited input fluorescent channels are available. When trained with DINO algorithm, ChannelViT consistently outperforms standard ViT on input images with varying sets of fluorescent channels [86]. Altogether, these findings underscored the formidable efficacy and robustness of SSL approaches in morphological profiling.

Most of the representation learning approaches described in this section have been benchmarked on the BBBC021 dataset with these evaluation metrics. Their performance is summarized in Table 3. From this comparison, WS-DINO, the weakly supervised method from Cross-Zamirski *et al*. [77] achieved the best performance. The transfer learning method from Ando *et al*. [37] and the self-supervised contrastive learning method from Perakis *et al*. [84] also showcased strong performance in learning meaningful phenotypic embeddings.

**Table 3:** Model performance comparison by MOA classification accuracy on the BBBC021 dataset

Approach	Description	NSC^a^	NSCB^b^	Drop^c^	Reference
Conventional feature engineering	CellProfiler with Factor Analysis	94%	77%	17%	Ljosa 2013 [36]
	CellProfiler with illumination correction	90%	85%	5%	Singh 2014 [71]
Supervised learning	CNN with Noisy-AND pooling function	96%	N/A	N/A	Kraus 2016 [69]
	Multiscale-CNN	93%	N/A	N/A	Godinez 2017 [70]
Transfer Learning	ImageNet Pretrained Inception-v3 with illumination correction and greyscale transformation	91%	N/A	N/A	Pawlowski 2016 [73]
	Pretrained Deep Metric Network with TVN postprocessing	96%	95%	1%	Ando 2017 [37]
Weakly supervised learning	Weakly supervised ResNet-18 with Mixup regularization	95%	89%	6%	Caicedo 2018 [38]
	WS-DINO finetuned on BBBC021 with compound as weak label	98%	96%	2%	Cross-Zamirski 2022 [77]
Self-supervised learning	CytoGAN (LSGAN)	68%	N/A	N/A	Goldsborough 2017 [46]
	VAE+	93%	82%	11%	Lafarge 2019 [47]
	UMM discovery with NSCB as best epoch criterion	95%	89%	6%	Janssens 2021 [81]
	Contrastive learning with whitening postprocessing	96%	95%	1%	Perakis 2021 [84]

^a^NSC (Not-Same-Compound matching accuracy).

^b^NSCB (Not-Same-Compound-and-Batch matching accuracy).

^c^Drop.

For deep learning approaches to achieve decent performance in morphological profile analysis, factors such as image dataset characteristics, model complexity, and computational resources must be carefully considered. Increasing the size and diversity of the training set, for example, by including image sets acquired from different laboratories, serves as an effective factor in enhancing performance, more so than simply increasing the model size [23]. In addition, applying appropriate image augmentations significantly benefits the performance of SSL methods such as DINO. Particularly, applying color augmentation on each fluorescent channel independently, through random brightness changes and intensity shifts, has been shown to produce the most significant positive impact on model performance [23]. In terms of computational time and costs, DINO with Graphics Processing Unit (GPU) acceleration can process and analyze data significantly faster than feature-based approaches, and despite requiring GPUs, it incurs lower infrastructure costs for analyzing per cell plate [23].

### Integrating morphological data in multimodal learning for drug discovery

With the advances in biotechnology, a wealth of data from various modalities can be generated and collected to facilitate drug discovery. Cheminformatics, for example, has made substantial contribution to drug discovery through analysis and representation of chemical structures and exploiting the similarity principle [87]. Chemical structure data of compounds are always readily available, and predicting compound bioactivity based on this data modality can be performed virtually. However, elucidating the intricate relationship between structure and biofunction is a challenging task [87]. On the other hand, ‘Omics’ profiles, such as genomics, transcriptomics, proteomics, and metabolomics, can characterize treatment outcomes from different aspects. However, assay cost and scalability emerge as major concerns for high-throughput studies [88]. Indeed, every modality of data utilized in the drug discovery presents its unique set of advantages and disadvantages. A detailed comparison is summarized in Table 4. Integrating these modalities is promising to maximize their potentials and mitigate the limitations, thereby providing a comprehensive understanding of treatment effects. Notably, recent research has shown that different data modalities, such as chemical structure, morphology, and gene expression, exhibit complementary strengths in predicting treatment effects [89]. Integrating morphological data with other data modalities using machine learning– or deep learning–based approaches has now become an active field of research.

**Table 4:** Comparison of transcriptomic and morphological profiling data for drug discovery

Attribute	Morphological profiling	Transcriptomic profiling
Infrastructure requirements	High-content imaging system. Some requires lab automation workflow.	Next-generation sequencer. Some requires cell-sorting capability.
Scalability	Scalable for Cell Painting assay.	Scalable for L1000 assay.
Cost	Low cost for conducting assays but high cost in system setup.	In general, low cost for newer platforms.
Data interpretability	Not interpretable on gene expression level.	Interpretable on gene expression level.
Data processing framework	Best practices for conventional feature-engineering approach have been made. Processes such as batch correction remain to be standardized.	Mostly standardized.
Reproducibility	Can be experimental platform dependent. Variations between data producing sites is non-trivial.	Technically reproducible. Biological reproducibility usually needs to be confirmed.

The integration of structural models with cell morphology models has been demonstrated to improve biological assay outcome prediction accuracy. Seal *et al.* proposed the similarity-based merger model, which combines the scaled predicted probabilities from individual models trained on Cell Painting images and chemical structures, and the morphological and structural similarities between test and training compounds [90]. Specifically, the predictions from individual models and similarity values are used to fit a logistic regression model to predict the test compound activity. The authors demonstrated that the similarity-based merger model outperforms soft-voting ensemble, hierarchical model, or either of the individual models trained on unimodal data [90].

In addition, SSL techniques such as contrastive learning approaches have also been utilized to align multimodal data sources to enrich morphological profiling analysis in drug discovery [91–93]. For example, a method known as Contrastive Leave One Out boost for Molecule Encoders (CLOOME) has been proposed, aiming to learn aligned representations derived from the compound’s chemical structure and the corresponding cellular images obtained after treatment with the same compound [91]. Its learning framework incorporates a microscopy image encoder, a molecule structure encoder, and uses the InfoLOOB objective [94] to learn the aligned embedding of treatment image and compound structure [91]. Similarly, Zheng *et al.* presented the Molecular graph and hIgh content imaGe Alignment (MIGA) framework with an image encoder and a graph neural network (GNN)–based structural encoder [93]. To align graph embeddings with image embeddings, three contrastive objectives are used: graph-image contrastive learning, masked graph modeling, and generative graph-image matching. The crossmodal representation learned with this framework improves performances on several downstream tasks [93]. This approach is extended further by Nguyen *et al.* to develop Molecule-Morphology Contrastive Pretraining (MoCoP) [92]. This framework uses a morphology encoder, a gated GNN (GGNN)–based molecule encoder, and the modified InfoNCE objective [95] to learn multimodal representation. The GGNN pretrained with MoCoP can be fine-tuned for downstream quantitative structure–activity relationship (QSAR) tasks [92]. Furthermore, active learning approach has been used to boost the performance of image-based and structure-based models and benefit the downstream QSAR tasks. The initial image-based and structure-based models assist selecting candidate compounds to be validated in toxicity assays. Once the wet-lab assays are completed, assay readouts will be collected as new annotations to continue refining both models. This iterative approach has been applied to detect compounds with mitochondrial toxicity [96].

In addition to chemical structure data, integrating transcriptomic profile with cell morphology serves as another crossmodal combination. A prevalent assay for obtaining gene expression profile is the L1000 assay [97]. Both Cell Painting and L1000 assays are scalable and provide complementary data. Compared to the transcriptomic profile from L1000, the morphological profile from Cell Painting is more reproducible yet susceptible to batch and well position effects. Conversely, L1000 captures more diverse features. Collectively, these two profiling modalities measure overlapping and assay-specific MOAs [98]. Besides the L1000 transcriptomic profile, another gene expression-based assay, Functional Signature Ontology (FUSION), can be fused with morphological profiling data to assign MOAs to complex natural product fractions in pair with metabolomic profiling data [99]. Comparative studies have shown that transcriptome-based and morphology-based models offer comparable or better performance in MOA prediction, compared to the chemical structure-based model [100]. These findings provide rationale and potential advantages of integrating transcriptomic and morphological profiling for drug discovery. More discussions on the applications and concerns of integrating these two data modalities have been recently characterized [101, 102]. Datasets with matched transcriptomic and morphological profiling data are summarized in Table 5.

**Table 5:** Multimodal datasets with matched transcriptomic and morphological profiling data

Cell type	Transcriptomic profiling description	Transcriptomic profile URL/Identifier	Morphological profile URL/Identifier	Reference
A549	L1000	https://figshare.com/articles/dataset/L1000_data_for_profiling_comparison/13181966/2	https://idr.github.io/idr0125-way-cellpainting/	Way 2022 [98]
A549	L1000	https://www.ncbi.nlm.nih.gov/geo/query/acc.cgi?acc=GSE83744	https://registry.opendata.aws/cell-painting-image-collection/	Haghighi 2022 [102]
A549	L1000	https://figshare.com/articles/dataset/L1000_data_for_profiling_comparison/13181966	https://zenodo.org/records/3928744#.YNu3WzZKheV	Haghighi 2022 [102]
U2OS	L1000	https://www.ncbi.nlm.nih.gov/geo/query/acc.cgi?acc=GSE92742	https://idr.openmicroscopy.org/webclient/?show=screen-1251	Haghighi 2022 [102]
U2OS	L1000	https://www.ncbi.nlm.nih.gov/geo/query/acc.cgi?acc=GSE92742	http://www.cellimagelibrary.org/pages/project_20269	Haghighi 2022 [102]
U2OS	L1000	https://github.com/carpenterlab/2017_rohban_elife/tree/master/input/TA-OE-L1000-B1	https://idr.openmicroscopy.org/webclient/?show=screen-1751	Haghighi 2022 [102]
Hela	FUSION	Upon request	Upon request	Hight 2022 [99]

**Figure 6:**
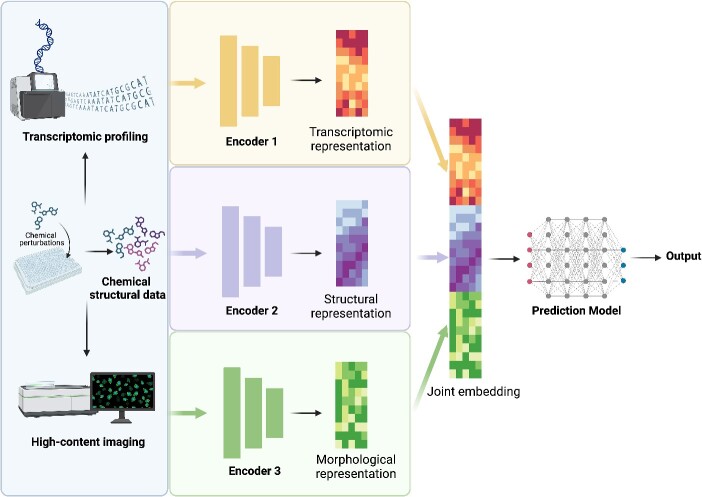
Combine morphological data with other data modalities. The image data obtained from morphological profiling assays can be combined with other modalities of profiling data to perform downstream tasks jointly. One strategy involves training individual models to extract representations from each data modality, such as image data, chemical structural data, and transcriptomic data. These individual representations contribute to a joint embedding, which is subsequently utilized for downstream analyses.

Data fusion methods have been widely used to integrate multimodal data (Fig. 6). In general, these methods can be categorized as early fusion and late fusion. Early fusion works by integrating the separate raw data modalities into a unified representation before feeding into the deep learning model for feature extraction. In contrast, late fusion combines the predictions of individual models, each built on a specific data modality. Algorithms such as cooperative learning have been proposed to enhance the alignment between predictions [103]. To integrate morphological, transcriptomic, and chemical structure profiles, Seal *et al.* compared both early and late fusion methods in detecting mitochondrial toxicity. They reported that the late fusion model can accurately determine the mitochondrial toxicity of compounds that have inconclusive toxicity results reported previously [104].

To identify perturbation effects in distinct feature space of morphological and transcriptomic data, Smith *et al*. proposed Perturbational Metric Learning (PeML) for similarity metric learning for multimodal data representation [105]. This WSL approach aims to learn an embedding to maximize the similarity between replicates, while non-replicates stay dissimilar. This learning methodology can be applied to both morphological and transcriptomic profiles and has demonstrated improved performance in MOA prediction [105].

Although the integration of morphological and transcriptomic (L1000) profiling offer benefits in MOA prediction, this orthogonal platform still faces challenges. These include limited resolution when identifying bioactive compounds that exhibit widespread cellular effects and reduced sensitivity when investigating bioactive compounds that do not induce distinct morphological changes [99]. To address these limitations, researchers have also investigated metabolomics-based approaches combining morphological characteristics to uncover changes in intracellular metabolism under various conditions [106]. Since metabolites in the cell can provide a comprehensive information of the cell state and define cellular phenotype in response to perturbations, combining cell morphology and metabolomics analysis has proven beneficial. For example, untargeted Mass Spectrometry (MS)–based metabolomics can be integrated with morphological profiling into a single platform to facilitate the quick identification and functional annotation of natural products in a high-throughput setting [99]. High-throughput image-based profiling pipeline can also be combined with multiparametric metabolic profiling approaches, such as oxygen consumption measurements and untargeted MS-based metabolomics to investigate the toxicity mechanism of the antiviral drug Tenofovir [107]. Furthermore, this combined approach can help optimizing microbial biosynthesis strategy, such as improving rapamycin production in *Streptomyces hygroscopicus* [108]. These studies underscore the significant advantages of integrating metabolomics and morphological, along with other data modalities in accelerating drug discovery process. With advances in MS techniques like MALDI-MS continuing to enhance throughput in metabolomic profiling [109], future studies will increasingly integrate these data with morphological profiling. Concurrently, development of these integrated platform calls for deep learning methods capable of facilitating multimodal learning using both morphological and metabolomics profiles.

In summary, applying deep learning approaches to integrate morphological data with other modalities, such as chemical structure, transcriptomic, and metabolomic data, demonstrates growing importance in drug discovery efforts. Techniques like contrastive learning and various data fusion methods are emerging to align multimodal data. The continuous curating of such multimodal datasets will further contribute to this burgeoning field.

## Novel applications of morphological profiling in drug discovery

Machine and deep learning approaches have significantly contributed to morphological profiling, enriching various aspects of phenotypic drug discovery. Applications such as identifying small-molecule MOAs, lead optimization, and predicting toxicology have been extensively reviewed elsewhere [110–113]. In the following sections, we will discuss the recent advances in several novel applications.

### Construct genotype–phenotype relationship and gene function network

Mapping genotype to disease-relevant phenotype has been a critical question in genomics. To address this challenge, genome-scale pooled CRISPR screens have been used to provide insights into gene functions. However, conventional screening readouts are relatively low in dimensionality (such as cell viability, proliferation, or expressions of biomarkers), thereby providing a constrained view of disease-relevant phenotype [114]. While high-content transcriptomic data from scRNA-seq can be measured from pooled CRISPR screens, the cost of achieving high-content readout as such from a genome-wide CRISPR screen can be unfeasibly high [114]. To overcome this hurdle, image-based profiling can provide high-content morphological readout for CRISPR screens at the genome scale [33, 115, 116]. Notably, optical-pooled CRISPR screens [117] can be combined with image-based profiling to create a genome-wide perturbation atlas and to construct a gene function network based on the uncovered genotype–phenotype relationships [115, 118, 119]. For example, Ramezani *et al.* developed a Cell Painting–based optical-pooled cell profiling approach (PERISCOPE) to allow pooled CRISPR screens to have high-dimensional cellular morphological profiles as endpoint readouts. This scalable pipeline has been applied to A549 cells and human cervical cancer cells (HeLa) to investigate gene knockout responses and identify gene clusters based on morphological similarity [118]. Sivanandan *et al.* introduced a similar technique termed Cell Painting Pooled Optical Screening in Human Cells (CellPaint-POSH). With this approach, a screening with a druggable genome library of 1640 genes has been conducted on A549 cells. Notably, this work applied the SSL DINO-ViT model (section Representation Learning for Morphological Profiling) for image representation and demonstrated decent performance in recovering the gene function network [119]. Such results further attest to the efficacy and robustness of deep learning approaches in generating informative image representations, subsequently leading to valuable biological insights.

In the efforts of mapping genotype to phenotype, the observation of “proximity bias” has been reported, whereby the phenotypes of CRISPR knockouts demonstrate higher similarity to biologically unrelated genomically proximal genes on the same chromosome arm than the biologically related genes. The cause of this artifact arises from widespread chromosome arm truncation due to Cas9 nuclease activity and is not observed in shRNA or CRISPR interference (CRISPRi) perturbations. Performing arm-based geometric normalization of features at gene level can reduce this bias without compromising the recovery of biological relationship [120].

### Characterize perturbation impacts in dynamics

An emerging advance of morphological profiling is toward live-cell phenotyping, which can be performed by fluorescent or phase-contrast imaging, and by continuous imaging [27, 121] or dynamic imaging [48]. Several advantages accompany this approach. First, adding temporal variables to the morphological profile improves assay predictive power [27]. For example, in a live-cell imaging-based profiling assay, a library of 1008 The United States Food and Drug Administration (FDA)-approved drugs with manual annotations was profiled against 15 reporter cell lines that expressed fluorescent protein–tagged organelle or pathway markers. The morphological profile was generated from 24-h high-content imaging and can be used to accurately infer 41 of 83 testable MOAs [27]. Beyond this, live-cell imaging enables the characterization of cell-state transition dynamics, a critical feature in developmental biology [48, 121]. Human pluripotent stem cells (hPSCs) coexpressing histone H2B and cell cycle reporters can be profiled in a multi-day, high-content manner at single-cell resolution. With this profile, a deep learning model can be trained to provide highly sensitive predictions of spatiotemporal single-cell fate dynamics, as early or even earlier than cell state–specific reporters [121]. Moreover, live-cell morphological features of human-induced pluripotent stem cells (hiPSCs) can even be used to predict differentiation marker gene expression [48]. This approach involves performing phase-contrast imaging and bulk RNA-sequencing at each consecutive passage of hiPSCs. A VAE variant, VQ-VAE [122], learns the image feature vector in a self-supervised approach. A number of Support Vector Regression (SVR) models, each corresponding to a differentiation marker, were trained to predict differentiation marker gene expression from the image feature vector. Bulk RNA-sequencing readouts were used as labels for this supervised learning process. Altogether, this approach builds the relationship between transcriptional and live-cell morphological profiles [48].

Deep learning models such as DynaMorph [123] and DEEP-MAP [121] have been proposed to analyze morphological profiles in dynamics. To take DynaMorph for example, VQ-VAE was trained to learn a representation of cell shape through a self-supervised image reconstruction auxiliary task. To ensure that cell shape changes smoothly between neighboring frames, a temporal matching loss was applied. The representation of cell shape regularized by the temporal continuity can distinguish morphodynamic states of microglia in response to pro- and anti-inflammatory stimuli [123].

### Guide *de novo* hit design

Although the typical downstream applications of morphological profiling have been focused on clustering or classification tasks (section An Overview of Image-Based Profiling Data Analysis), Zapata *et al*. proposed to leverage morphological profiles to guide *de novo* molecular design with GANs [124]. Compared to using transcriptional profiling for compound *de novo* design [125], morphological profiling provides higher throughput with less cost. More importantly, more than 40% of the generated molecules have drug-like physicochemical properties, and more than half are expected to be synthesizable. This model can also be generalized to morphological profile with genetic perturbations such as gene overexpression. These findings indicate that this approach is able to effectively translate morphological similarity into chemical similarity with high efficiency [124].

### Facilitate image-based profiling in advanced biological models

Organoids are hetero-cellular biomimetic tissue models that have become a powerful experimental tool transforming basic science and translational research [126]. While the traditional low-throughput methods provide valuable biological insights, high-throughput methods are needed to fully exploit the potential of organoids as *ex vivo* models. Modeling the development of disease with organoids that can recapitulate tissue structure, pathology, phenotypes, and differentiation has revolutionized the study of various human diseases including cancer [126, 127]. In a recent study, Silva *et al.* and Atanasova *et al.* demonstrated the effect of small molecules in mouse pancreatic acinar that causes inhibition or reversal of acinar-to-acinar ductal metaplasia (ADM) using high-content image-based screening in organoid culture [128, 129]. Advances in technology in organoid culture and the remarkable self-organizing properties reflecting key structural and functional attributes of organs such as brain, kidney, lung, gut, or similar even hold promise to predict drug response in a personalized fashion.

While organoids are normally cultured in bulk in an extracellular matrix, these bulk cultures can physically overlap, which makes it challenging to track the growth and properties of individual organoids in high-throughput assays. Various microwell designs have been introduced to overcome specific challenges associated with image-based analysis but still struggle with large numbers of organoids [130, 131]. Using different organoid culture methods, phenotypic assays can be designed using features like whole organoid morphology, growth rates, or movement with simple brightfield imaging. Many of these methods rely on cellular aggregation to generate spheroids rather than growing organoids from single cells [132–134]. These can cause limitations in understanding the phenotypic heterogeneity, while most of the methods do not employ integrated analytical pipelines into the overall workflow [135–139] or the ability to selectively retrieve organoids for downstream investigations. Overcoming some of these issues, Forsyth and a team of researchers [126] have built an open-source microwell-based platform for high-throughput quantification using image-based parameters. The method utilizes an organoid-optimized deep-learning model that can be integrated with existing culturing protocols and micro-well platforms to investigate phenotypic features across different tissues. Additionally, patient-derived tumor organoids have been developed into powerful organoid-based discovery platforms in recently demonstrated using CRISPR-Cas9 screening for patient-specific functional genomics [140]. Defined mutations are introduced to transform normal organoids to tumorigenic growth upon xenotransplantation, combining the exploratory power of CRISPR-Cas9 screening with 3D organoids [133, 136, 141]. These advances demonstrate that organoids are powerful experimental models for morphological profiling to study the maturation and progression of various diseases.

### Enable natural product–based drug discovery

Natural products (NPs) and their structural analogues have made a major contribution to pharmacotherapy, playing a key role in drug discovery [3, 4]. Recent years have witnessed that AI approaches have substantially advanced the efficient identification of drug candidates from NPs, marking notable progress in drug discovery [5]. NP-based drug leads are typically identified by phenotypic assays [4]. To that end, an image-based profiling platform has been developed to study toxicity, structure–activity relationship (SAR), MOA, and potential off-target effects of NPs [6]. For example, a high-throughput screening on MIN6 β cells with 6298 marine NP fractions has been performed to select for hit compounds with nontoxic and long-lasting effects in inhibiting glucose-stimulated insulin secretion [142]. In combination with MS analyses and NMR analyses, aureolic acid CMA2 has been identified as the major component of the top hit fraction derived from *S. anulatus*. Treating MIN6 cells with CMA2 leads to decreased nuclei counts determine by the 4′-6-Diamidino-2-phenylindole (DAPI) staining, attesting to its bioactivity [143]. In another study, botanical NP extracts have been screened for blockade of SARS-CoV-2 infection in human 293TAT cells. A leading hit, the extracts of *S. tetrandra*, is further investigated on its antiviral MOA through phenotypic assays based on intracellular phospholipids formation [144]. In addition, high-dimensional phenotypic readouts also assist exploring NP MOA. To understand the MOA of the Polyketide Lagriamide B from the Burkholderiales strain, its morphological impact on U2OS cells is investigated through the Cell Painting assay followed by high-content imaging. At low treatment concentration, Lagriamide B leads to disruption in actin polymerization and incomplete cytokinesis, and at high concentration, low cell count and decreased cell size are observed. These phenotypic effects indicate an MOA of Lagriamide B in actin polymerization disruption [145].

Specifically, integrating morphological with multi-omics profiling helps annotate the bioactive components of NPs, which addresses one of the most significant challenges in NP-based drug discovery [99]. For example, an integrated framework of morphological and transcriptomic profiles has been used to annotate marine bacteria extracts based on its untargeted metabolomics profile [99]. This orthogonal platform demonstrated a new paradigm to understand the association between NP components and treatment phenotypes and underscored the importance of integrating multimodal profiling data for drug discovery (section Integrating Morphological Data in Multimodal Learning for Drug Discovery).

## Challenges and outlook

Morphological profiling is poised to have a profound and continuing impact on phenotypic drug discovery in the next decade and beyond. Deep learning approaches will continue to empower morphological profiling with enhanced accuracy and efficiency [110]. However, several challenges await resolution in order to fully leverage cellular images as a reliable and insightful resource, as will be discussed in this section.

Although representation learning (section Representation Learning for Morphological Profiling) has become a robust approach to learn cellular features with less manual input than the conventional feature-engineering approach, it is susceptible to confounding factors such as batch effects. Batch effects are variations in data caused by the differences in the technical execution of each experimental batch. Such confounding factors introduce irrelevant sources of variation into data and can potentially mislead biological conclusions [146]. Disentangling these confounding factors from phenotypes is a crucial step to recover a true biological signal. Significant progress has been made in this regard, with methods such as TVN [37], BEN [147], TEAMS [148], CDCL [80], and GRU-based regularization [38]. Furthermore, batch correction methods for transcriptomic profiles may be applicable. A recent study on subsets of JUMP-CP demonstrated that Harmony, a non-linear method developed for processing scRNA-seq data, consistently outperforms other transcriptomic profile batch correction strategies in balancing batch removal and biological variation conservation [149]. In addition to the aforementioned methods, adding a context token to include batch-specific information during image representation learning also demonstrated decent performance in out-of-distribution generalization and batch variation handling [150]. To evaluate and compare batch correction strategies, RxRx1, a Cell Painting image dataset of genetic perturbations with 51 experimental batches from four cell types, has been systematically designed [146]. With the development and sharing of the benchmarked dataset, future work will continue to enhance upon existing methods. Improved handling of the confounding factors will further facilitate data sharing and reproducibility between data generation sites, thereby bringing significant benefits to the broader scientific community.

The success of phenotypic drug discovery heavily relies on disease relevance of the biological model. Applying relevant cell types and perturbations in morphological profiling assay is essential, but not sufficient to guarantee translatability [1]. Recent efforts have been made to apply increasingly multiplex biological model systems for image-based profiling, such as cocultured 2D cell lines [151] and 3D organoids [152, 153]. However, on the computational side, most approaches have been built upon Cell Painting assay images from mono-cultured 2D cells. Therefore, many challenges remain in generalizing these approaches to a multiplex biological model. For example, how do current cell segmentation (section Deep Learning–Facilitated Cell Segmentation for Image Analysis) and representation learning methods (section Representation Learning for Morphological Profiling) perform on 3D images? How is the quantity and quality of 3D image dataset that can be utilized for effectively training for fine-tuning deep learning models? How generalizable are the representation learning frameworks (section Representation Learning for Morphological Profiling) to cellular images consisting of multiple cell types, each demonstrating different morphology? How to integrate morphological data and other modalities of data (section Integrating Morphological Data in Multimodal Learning for Drug Discovery) from a multiplexed cell system to obtain cell–cell interaction information? Overcoming these hurdles will bring morphological profiling to the next level of clinical translatability.

In terms of integrating morphological profile with omics data (section Integrating Morphological Data in Multimodal Learning for Drug Discovery), compared to bulk transcriptomic readouts, single-cell transcriptomics, spatial transcriptomics and translatomics offer a wealth of gene expression information at individual cellular and subcellular levels [154–157]. Advances such as sci-RNA-seq3 have enabled single-cell transcriptional profiling in high throughput [158]. Given this technical progress, future work may establish an orthogonal profiling platform to combine morphological and single-cell profiling, thereby linking molecular phenotype to cellular phenotype at single-cell resolution. In addition, integrating Perturb-seq with image-based profiling will become a promising future direction to characterize the impact of genetic perturbations with single-cell transcriptomics and morphological readouts [159]. High-quality datasets of such should be established to encourage the development and evaluation of data integration approaches.

Last but not least, despite the impressive inferential capabilities of deep learning approaches, drawbacks remain that the explainability of these ‘black box’ models is unsatisfying [160]. In drug discovery especially, model interpretability is important to ensure that the biological conclusions are valid. To mitigate this, several efforts have been initiated to improve model interpretation in morphological profiling. For example, Chow *et al.* trained VAEs to interpret latent space feature representations in Cell Painting assay [161]. In the broader field of computer vision, techniques such as class activation mapping [162, 163] have been proposed to provide visual explanations for deep neural networks. Future work should continue to develop or advance techniques as such to morphological profiling to enhance model interpretability [162].

## Concluding remarks

Morphological profiling represents a powerful, high-throughput, data-intensive, and cost-efficient technique for phenotypic drug discovery. It offers an unbiased and high-dimensional image readout of cellular phenotype in response to various perturbations, thereby providing a comprehensive view on compound bioactivity. Emerging techniques from computational biology and deep learning communities have made significant progress in enhancing the analytical pipeline from representation to prediction. While challenges remain in this fast-evolving field, future work will continue to coordinate multidisciplinary efforts in leveraging visual phenotypes to empower drug discovery.

**Key Points**

- Image-based profiling is a valuable tool in phenotypic drug discovery and facilitates understanding cell biology in response to different perturbations.
- Deep learning approaches have contributed significantly to morphological profiling data analysis through segmenting cellular images, learning robust image representations, and integrating morphological data with other data modalities.
- These advancements enable many novel downstream applications, such as constructing gene function network to map genotype–phenotype relationship, characterizing perturbation impacts in dynamics, guiding *de novo* hit design, identifying compound MOAs in 3D organoid model, and enabling natural product-based drug discovery.
- Innovative solutions are needed in several challenging aspects, such as handling batch effects, analyzing multiplex biological model, integrating with spatial-omics, and improving model interpretability.

## Data Availability

Details about the data discussed in this study have been incorporated in the article. No additional data were generated for this study.

## Author Biographies

**Qiaosi Tang** is a scientist at Calico Life Sciences, an Alphabet-founded research lab for human aging research. Her research focuses on using morphological profiling and machine learning methods to reveal age-related disease phenotype and identify potential therapeutic targets.

**Ranjala Ratnayake** is a Research Associate Professor and Assistant Director of Center for Natural Products, Drug Discovery and Development (CNPD3), in the Department of Medicinal Chemistry at the College of Pharmacy, University of Florida. Her research focuses on using multidimensional screening platforms to identify novel drug therapies from complex natural product libraries.

**Gustavo Seabra** is a Research Associate Professor in the Department of Medicinal Chemistry and the Center for Natural Products, Drug Discovery and Development (CNPD3) at the College of Pharmacy, University of Florida. His research focuses on the development and application of diverse computational chemistry methods and machine learning methods for molecular modeling.

**Zhe Jiang** is an Assistant Professor in Computer & Information Science & Engineering (CISE) at the University of Florida. His lab’s mission is to design and develop accurate, scalable, and robust AI and machine learning algorithms and tools inspired by interdisciplinary applications such as biomedicine.

**Ruogu Fang** is an Associate Professor and Pruitt Family Endowed Faculty Fellow in the J. Crayton Pruitt Family Department of Biomedical Engineering at the University of Florida. Her group’s research revolves around the integration of artificial intelligence (AI) and deep learning with the intricacies of the human brain.

**Lina Cui** is an Associate Professor in the Department of Medicinal Chemistry at the College of Pharmacy, University of Florida. Her group develops and applies molecular tools for molecular imaging in various collaborative projects to explore the progression of diabetes, neurodegeneration, and other age-related diseases.

**Yousong Ding** is an Associate Professor in the Department of Medicinal Chemistry at the College of Pharmacy, University of Florida. His research interests include natural product biosynthesis, drug discovery and development, synthetic biology, protein engineering, and chemical biology.

**Tamer Kahveci** is a Professor and Associate Chair of Academic Affairs in the Computer and Information Science and Engineering Department at the University of Florida, serving as the Associate Chair of Academic Affairs. His research focuses on bioinformatics and has worked on indexing sequence and protein structure databases, sequence alignment, and computational analysis of biological pathways.

**Jiang Bian** is a Professor and Division Director of Biomedical Informatics at the University of Florida and Chief Data Scientist and Chief Research Information officer of UF Health. His research focuses on data-driven medicine and development of novel informatics methods, tools, and systems to support clinical and clinical research activities.

**Chenglong Li** is the Nicholas Bodor Professor in Drug Discovery and Professor in the Department of Medicinal Chemistry at the College of Pharmacy, University of Florida. He is also the Director of the NIGMS-supported CBI (Chemistry-Biology Interface) Graduate Training Program at the University of Florida. His research focuses on molecular recognition, with a strong application to structure-based computer-aided drug design.

**Hendrik Luesch** is a Professor in the Department of Medicinal Chemistry, the endowed Debbie and Sylvia DeSantis Chair in Natural Products Drug Discovery and Development, and the founding Director of Center for Natural Products, Drug Discovery and Development (CNPD3) at the College of Pharmacy, University of Florida. His multidisciplinary research program at the interface of chemistry and biology integrates genome mining, synthetic and chemical biology, isolation and spectroscopic structure determination, chemical synthesis, target identification, and mechanism of action studies, followed by the preclinical development of candidate molecules.

**Yanjun Li** is an Assistant Professor (AI Initiative) in the Department of Medicinal Chemistry at the College of Pharmacy, University of Florida. His research interests span the fields of deep learning, AI-driven drug discovery, and computational biology, with a particular emphasis on deep generative modeling for *de novo* molecule design and geometric deep learning for molecular recognition.
